# Comparative Study of 150 vs. 200 Units of Botulinum Toxin as Treatment for Vaginismus

**DOI:** 10.1055/s-0042-1751287

**Published:** 2022-07-11

**Authors:** Zeena R. Helmi

**Affiliations:** 1 Department of Gynecology and Obstetrics, College of Medicine, Mustansiriyah University, Baghdad, Iraq

**Keywords:** vaginismus, botulinum toxin, sexual pain, anxiety, pain treatment, vaginismo, toxina botulínica, dor sexual, ansiedade, tratamento da dor

## Abstract

**Objective** To comparatively evaluate the outcome of treatment with 150 versus 200 units (U) of botulinum toxin in achieving pain-free intercourse and relieving muscle contraction in order to allow gynecological examination.

**Methods** In this comparative prospective observational study, 99 patients with vaginismus were treated with botulinum toxin injections from September 2016 to August 2021. Diagnosis and grading of vaginismus severity were assessed using a Female Sexual Function Index (FSFI) questionnaire. Under local or general anesthesia, botulinum toxin diluted with preservative-free saline (150 U and 200 U) was injected into, above, and below the right and left bulbospongiosus muscle and the lateral submucosal areas of the introitus and perineal body using an insulin syringe. Patients were recalled after 2 weeks, and the postoperative outcome was recorded using a similar preoperative questionnaire.

**Results** Overall, the mean age of patients was 30.2 years. The baseline and clinical characteristics were comparable between the 2 groups ( *p* > 0.05). Significant improvements were seen in the pain and anxiety scores of finger penetration, dilator use, intercourse, and cotton swab in individual groups. The intergroup comparisons between 150 U and 200 U of Botox were not statistically significant ( *p* > 0.05).

**Conclusion** Low-dose Botox (150 U) is equally effective as high dose Botox injections (200 U) in vaginismus patients. Therefore, Botox-150 U can be used to treat vaginismus as an alternative to high doses of the same substance.

## Resumo

**Objetivo** Avaliar comparativamente o resultado do tratamento com 150 versus 200 unidades (U) de toxina botulínica na obtenção de relações sexuais sem dor e no alívio da contração muscular para permitir o exame ginecológico.

**Métodos** Neste estudo observacional prospectivo comparativo, 99 pacientes com vaginismo foram tratadas com injeções de toxina botulínica de setembro de 2016 a agosto de 2021. O diagnóstico e a classificação da gravidade do vaginismo foram avaliados usando um questionário Female Sexual Function Index (FSFI). Sob anestesia local ou geral, injetou-se toxina botulínica diluída em soro fisiológico sem conservantes (150 U e 200 U) nos músculos bulbo esponjoso direito e esquerdo e nas áreas submucosas laterais do intróito e corpo perineal, utilizando-se uma seringa de insulina. Os pacientes foram chamados após 2 semanas, e o resultado pós-operatório foi registrado usando um questionário pré-operatório semelhante.

**Resultados** No geral, a média de idade dos pacientes foi de 30,2 anos. As características basais e clínicas foram comparáveis entre os 2 grupos ( *p* > 0,05). Melhorias significativas foram observadas nos escores de dor e ansiedade à penetração com dedo, uso de dilatador, relação sexual e cotonete em grupos individuais. As comparações intergrupos entre 150 U e 200 U Botox foram não estatisticamente significativas ( *p* > 0,05).

**Conclusão** Botox de baixa dose (150 U) é tão eficaz quanto injeções de Botox de alta dose (200 U) em pacientes com vaginismo. Portanto, o Botox-150 U pode ser usado para tratar o vaginismo como alternativa às altas doses da mesma substância.

## Introduction

Marion Sims, in 1862, used the term vaginismus for the first time to describe recurrent and persistent involuntary contractions of perineal muscles during penetration, resulting in anxiety, fear, and pain. 1 The prevalence of vaginismus varies between developed and developing countries due to cultural differences and ranges from 1 to 60%. 2 3 4 The American Psychiatric Society has defined vaginismus as “an uncontrollable contraction of the muscle of one-third of the external vagina that prevents intercourse.” 5 Currently, vaginismus is categorized under the category of genito-pelvic pain/penetration disorder as per the Diagnostic and Statistical Manual of Mental Disorders (DSM 5). 5 6 The World Health Organization, in its International Statistical Classification of Diseases and Related Health Problems, (ICD-11), has emphasized the pain associated with vaginismus and categorized it as a disorder of female sexual penetration pain. 7

Although various theories and multiple predisposing factors for vaginismus have been proposed, including negative psychological components on sex such as fear of first-time sex, sexual myths, anxiety during gynecological examinations, history of sexual and physical abuse, upbringing in a conflicting family, religious prejudice, cultural taboos, and sinful context lack of sex education, the exact etiology of vaginismus remains elusive. Moreover, factors including sexually transmitted infection, endometriosis, congenital abnormalities such as hymen anomaly, scarring from trauma, vaginal atrophy, pelvic inflammatory disease, and cancer may also induce vaginismus. 6 8

Vaginismus can be classified according to the International Classification of Diseases 11th edition (ICD-11) and the Diagnostic and Statistical Manual (DSM-V) as either primary, when a woman has never had intercourse, or secondary or acquired, when a woman loses the ability to have intercourse, usually as a result of acquired dyspareunia secondary to physical events, mental and psychological factors, infection or menopausal changes. Furthermore, vaginismus is classified as total vaginismus, where intercourse is not possible, and partial vaginismus, in which intercourse is possible but causes genital pain. 5 7 9 10 Repeated episodes of vaginismus and failed intercourse affect the sexual health and wellbeing of a woman, decrease self-confidence, affect marital relationships and outside-home relationships, and lead to infidelity and divorce. As procreation defines marriage and healthy sexual life, vaginismus affects a couple's emotional health within their marriage; thus, the treatment and improvement of vaginismus are of chief importance. 3 11

As vaginismus contributes to vaginal atrophy and dyspareunia, treating the same to achieve painless and satisfactory intercourse may be beneficial. Considering the physical and behavioral factors, various treatment modalities include cognitive and behavioral therapies, relaxation methods, sex education, vaginal dilation, vaginal desensitization methods like botulinum toxin, local anesthetic agents, psychotherapy or sex therapy, and physiotherapy of pelvic muscles; other invasive methods are ultrasound-guided inter-fascial plane blocks electromyography and biofeedback hypnotherapy. While mild vaginismus may respond to modalities and conventional therapies, severe forms require interventional therapies, like botulinum toxin injections. However, due to the variable efficacies of different interventions, no standard approach has been proposed to date. 4 6 10

Botulinum neurotoxin type A is a polypeptide that inhibits acetylcholine-associated muscular contraction and decreases muscle spasms and pain through various mechanisms. Additionally, it also reduces peripheral and central sensitization, Following its use in vaginismus in 1997, many researchers have explored the use of botulinum toxin in the treatment of vaginismus and have conferred that treatment with botulinum toxin is a safe and effective modality that inhibits vaginal muscle spasm and helps to achieve painless intercourse. 12 However, limited studies are available comparing the efficacy of different Botox doses in the treatment of vaginismus; thus, we sought to comparatively evaluate the outcome of the treatment with 150 vs 200 units (U) of botulinum toxin in achieving pain-free intercourse and relieving muscle contraction in order to allow gynecological examination.

## Methods

**Fig. 1:**
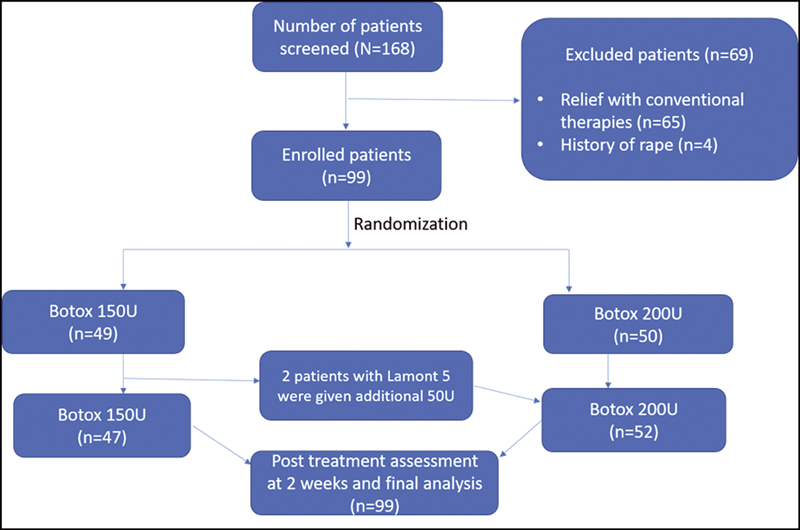
Fluxogram of participants in the study.

We conducted a comparative prospective observational study from September 2016 to August 2021 at an obstetrics and gynecology clinic at university hospital to evaluate the treatment efficacy of botulinum toxin among 99 patients with vaginismus. We obtained an ethical committee clearance from Mustansiriyha University (Ref. no. MOG 128; dated 2/2021), and the study was conducted in accordance with the declaration of Helsinki. The benefits and risks of botulinum toxin injections and procedures were explained to the patient, and a signed informed consent was obtained. All patients who contacted the clinic through social media and those referred by clinicians or relatives for painful intercourse and/or impossible intercourse with no relief from conventional therapies were included in the study. Patients with a history of sexual abuse or rape were excluded and referred for psychological counseling. Patient disposition and study design are depicted in Fig. 1 .

We recorded the demographic characteristics of patients, including age, occupation, employment status, and duration of complaint. According to the International Classification of Diseases 11th edition (ICD-11) and the Diagnostic and Statistical Manual (DSM-V) 5 7 the patients were categorized into primary and secondary types with a definitive diagnosis of vaginismus. All patients were given a Female Sexual Function Index (FSFI) questionnaire. 13 14 The questionnaire consisted of separate pain and anxiety grading for finger penetration, dilator use, cotton swab, and intercourse. The responses were graded as 1 = no pain or anxiety; 2 = moderately uncomfortable, 10 = impossible or extremely painful/severe anxiety. The patients were randomly allocated to receive either 150 U or 200 U of Botox treatment.

### Botox Injection Procedure

Before the treatment, one vial of frozen Botox containing 100 U was diluted with 2 mL of preservative-free saline without shaking to prevent foam formation. The procedure was conducted either under local or general anesthesia based on the patient's comfort. In the Botox 150 U group, Botox 50 U (1 mL) each was injected into the bulbospongiosus muscle and the lateral submucosal areas of the introitus, marked by the residual hymenal fragments, at 9'clock and 3 o'clock positions using a 1 mL insulin syringe. In the Botox 200 U group, 75 U (1mL) was used and injected into the areas similar to the Botox 150 U group. In patients with persistent spasm and tightness of the levator ani muscle, an additional 50 U was injected subcutaneously into the perineal body in both groups. To avoid urinary and rectal incontinence, care was taken not to inject the solution anteriorly or posteriorly. In some patients, an additional hymenectomy was done. Following injections, a medium-sized plastic speculum was inserted into the vagina for 6 hours. The patients were initially followed up after 2 weeks, and the postoperative outcome was recorded using a similar preoperative questionnaire. All patients were followed up for a year over phone calls.

### Statistical Analysis

Baseline demographic and clinical characteristics, Lamont-Pacik classification, and the recorded pre and postprocedure questionnaire responses were entered into a Microsoft Excel spreadsheet (Microsoft Corp., Redmond, WA, USA) and statistically analyzed using the IBM SPSS Statistics for Windows, version 20.0 (IBM Corp., Armonk, NY, USA). Continuous variables were presented as mean and standard deviation, while categorical variables were presented as frequency and percentages. Intergroup and intragroup comparisons of pre and postpain and anxiety scores were tested using the chi-squared or Fisher exact test. A *p* -value of < 0.05 was considered statistically significant.

## Results

We recruited 99 patients with an age range of 15 to 67 for the treatment (Botox 150 mg, *n* = 47; Botox 200 mg, *n* = 52). Intergroup comparisons of demographic and baseline clinical characteristics, outcomes, and complications are summarized in Table 1 .

**Table 1:** Baseline demographic and clinical characteristics

Variable	Botox 150 U N = 47	Botox 200 U N = 52	All patients N = 99	*p* -value
Age in years				
Mean (SD)	31.5 (9.8)	29.0 (6.6)	30.2 (8.3)	
Median (min–max)	28 (17–67)	28 (15–45)	28 (15–67)	
Occupation n (%)				0.611
Housewife	42 (89.4)	48 (92.3)	90 (90.9)	
Employed	5 (10.6)	4 (7.7)	9 (9.1)	
Residency				0.383
Baghdad	41 (87.2)	42 (80.8)	83 (83.8)	
Other governorate	6 (12.8)	10 (19.2)	16 (16.2)	
Gravid				0.104
0	44 (93.6)	47 (90.4)	91 (91.9)	
1	1 (2.1)	5 (9.6)	6 (6.1)	
3	2 (4.3)	0	2 (2)	
Para				0.068
0	44 (93.6)	46 (88.5)	90 (90.9)	
1	1 (2.1)	6 (11.5)	7 (7.1)	
3	2 (4.3)	0	2(2)	
Type				0.887
Primary	42 (89.4)	46 (88.5)	88 (88.9)	
Secondary	5 (10.6)	6 (11.5)	11 (11.1)	
Duration in months				
Mean (SD)	19.3 (18.9)	19.3 (21.8)	19.3 (20.4)	
Median (min–max)	12 (< 1–72)	12 (3–96)	12 (< 1–96)	
Anesthesia				0.135
Local anesthesia	4 (8.5)	1 (1.9)	5 (5.1)	
General anesthesia	43 (91.5)	51 (98.1)	94 (94.9)	
Finger penetration anxiety				0.586
1	0	0	0	
5	15 (31.9)	14 (26.9)	29 (29.3)	
10	32 (68.1)	38 (73.1)	70 (70.7)	
Finger penetration pain				0.586
1	0	0	0	
5	13 (27.7)	17 (32.7)	30 (30.3)	
10	34 (72.3)	35 (67.3)	69 (69.7)	
Dilator anxiety				0.494
NA	7 (14.9)	4 (7.7)	11 (11.1)	
1	0	0	0	
5	13 (27.7)	14 (26.9)	27 (27.3)	
10	27 (57.4)	34 (65.4)	61 (61.6)	
Dilator pain				0.171
NA	7 (14.9)	4 (7.7)	11 (11.1)	
1	0	3 (5.8)	3 (3)	
5	14 (29.8)	11 (21.2)	25 (25.3)	
10	26 (55.3)	34 (65.4)	60 (60.6)	
CS anxiety				0.002
NA	0	0	0	
1	22 (46.8)	9 (17.3)	31 (31.3)	
5	9 (19.1)	25 (48.1)	34 (34.3)	
10	16 (34)	18 (34.6)	34 (34.3)	
CS pain				0.327
NA	1 (2.1)	0	1 (1)	
1	38 (80.9)	37 (71.2)	75 (75.8)	
5	4 (8.5)	10 (19.2)	14 (14.1)	
10	4 (8.5)	5 (9.6)	9 (9.1)	
Intercourse anxiety				0.455
NA	0	1 (1.9)	1 (1)	
1	0	0	0	
5	13 (27.7)	18 (34.6)	31 (31.3)	
10	34 (72.3)	33 (63.5)	67 (67.7)	
Intercourse pain				
NA	0	1 (1.9)	1 (1)	
1	0	2 (3.8)	2 (2)	
5	8 (17)	6 (11.5)	14 (14.1)	
10	39 (83)	43 (82.7)	82 (82.8)	
GE anxiety				0.067
NA	0	3 (5.8)	3 (3)	
1	0	3 (5.8)	3 (3)	
5	13 (27.7)	18 (34.6)	31 (31.3)	
10	34 (72.3)	28 (53.8)	62 (62.6)	
GE pain				0.020
NA	0	3 (5.8)	3 (3)	
1	0	3 (5.8)	3 (3)	
5	4 (8.5)	11 (21.2)	1 (15.2)	
10	43 (91.5)	35 (67.3)	78 (78.8)	

Abbreviations: CS, cotton swab; GE, gynecological examination; n, number; NA, not available; SD, standard deviation; U, unit.

Overall, the mean (standard deviation; SD) age of the patients was 30.2(8.3) and was similar between both groups. Most of the patients (90.9%) in the study cohort were housewives residing in Baghdad (83.8%). There was no significant difference between occupation ( *p* = 0.611) and residency ( *p* = 0.383) among the 2 groups. The overall mean duration of the vaginismus was 19.3 (20.4) months. The frequency of primary vaginismus was higher than the secondary vaginismus (88.9% versus 11.1%). Type of vaginismus ( *p* = 0.887), gravid ( *p* = 0.104), para ( *p* = 0.068), and duration of vaginismus were comparable between the groups. Most of the patients underwent Botox treatment under general anesthesia (94.9%). In the preoperative intergroup comparisons of the questionnaire, except for cotton swab anxiety ( *p* = 0.002) and gynecological examination pain scores ( *p* = 0.020), no significant difference was seen in the anxiety and pain grades during finger penetration, dilator cotton swab, and intercourse tip penetrations ( *p* > 0.05) ( Table 1 ). In the post-treatment questionnaire, except for dilator pain scores ( *p* = 0.015), the pain and anxiety scores of all other variables in both treatment groups were similar ( *p* > 0.05). Overall, the improved outcome was seen in 91.9% of patients; no significant difference was observed between the two groups (91.5% versus 92.3%; *p* = 0.881). Around 80.8% of patients reported no side effects post-treatment. Between the groups, urinary and anal incontinence were seen in 17 and 2 patients, respectively, with no statistical difference ( *p* = 0.997) ( Table 2 ).

**Table 2:** Intergroup comparisons of posttreatment questionnaire response, outcome, and side effects,

Variables	Botox 150 U N = 47	Botox 200 U N = 52	All patients N = 99	*p* -value
Finger penetration anxiety				0.429
1	32 (68.1)	31 (59.6)	63 (63.6)	
5	10 (21.3)	17 (32.7)	27 (27.3)	
10	5 (10.6)	4 (7.7)	9 (9.1)	
Finger penetration pain				0.563
1	42 (89.4)	48 (92.3)	90 (90.9)	
5	4 (8.5)	4 (7.7)	8 (8.1)	
10	1 (2.1)	0	1 (1)	
Dilator anxiety				0.056
1	29 (61.7)	26 (50)	55 (55.6)	
5	11 (23.4)	23 (44.2)	34 (34.3)	
10	7 (14.9)	3 (5.8)	10 (10.1)	
Dilator pain				0.015
1	42 (89.4)	45 (86.5)	87 (87.9)	
5	1 (2.1)	7 (13.5)	8 (8.1)	
10	4 (8.5)	0	4 (4)	
CS anxiety				0.052
1	47 (100)	48 (92.3)	95 (96)	
5	0	4 (7.7)	4 (4)	
10	0	0	0	
CS pain				
1	47 (100)	52 (100)	99 (100)	−
5	0	0	0	
10	0	0	0	
Intercourse anxiety				0.364
1	30 (63.8)	32 (61.5)	62 (62.6)	
5	10 (21.3)	16 (30.8)	26 (26.3)	
10	7 (14.9)	4 (7.7)	11 (11.1)	
Intercourse pain				
1	39 (83)	44 (84.6)	83 (83.8)	
5	1 (2.1)	4 (7.7)	5 (5.1)	
10	7 (14.9)	4 (7.7)	11 (11.1)	
Outcome				0.881
Improved	43(91.5)	48 (92.3)	91 (91.9)	
Failed	4 (8.5)	4 (7.7)	8 (8.1)	
Side effects				0.997
None	38 (80.9)	42 (80.8)	80 (80.8)	
Urinary incontinence	8 (17)	9 (17.3)	17 (17.2)	
Anal incontinence	1 (2.1)	1 (1.9)	2 (2)	

Abbreviations: CS, cotton swab; n, number; U, unit.

Overall, significant improvement in the finger penetration pain and anxiety scores, dilator anxiety and pain scores, and intercourse anxiety scores were reported ( *p* < 0.05). Significant improvement in the finger penetration anxiety scores ( *p* = 0.037), dilator anxiety ( *p* = 0.000), dilator pain ( *p* = 0.001), intercourse tip anxiety ( *p* = 0.035) were reported in the 150 U Botox treatment group. In the 200U Botox treatment group, significant improvement was seen in finger penetration anxiety ( *p* = 0.02), dilator anxiety (0.000), and intercourse anxiety scores ( *p* = 0.031) scores ( Tables 3 , 4 and 5 ).

**Table 3:** Overall improvement in the study group

Variables	Pretreatment ( *n* = 99)	Posttreatment ( *n* = 99)	*p* -value
Finger penetration anxiety			0.000
1	0	63 (63.6)	
5	29 (29.3)	27 (27.3)	
10	70 (70.7)	9 (9.1)	
Finger penetration pain			0.116
1	0	90 (90.9)	
5	30 (30.3)	8 (8.1)	
10	69 (69.7)	1 (1)	
Dilator anxiety			0.000
NA	11 (11.1)	0	
1	0	55 (55.6)	
5	27 (27.3)	34 (34.3)	
10	61 (61.6)	10 (10.1)	
Dilator pain			0.003
NA	11 (11.1)	0	
1	3 (3)	87 (87.9)	
5	25 (25.3)	8 (8.1)	
10	60 (60.6)	4 (4)	
CS anxiety			0.387
1	31 (31.3)	95 (96)	
5	34 (34.3)	4 (4)	
10	34 (34.3)	0	
CS pain			Not computed
NA	1 (1)	0	
1	75 (75.8)	99 (100)	
5	14 (14.1)	0	
10	9 (9.1)	0	
Intercourse anxiety			0.002
NA	1 (1)	0	
1	0	62 (62.6)	
5	31 (31.3)	26 (26.3)	
10	67 (67.7)	11 (11.1)	
Intercourse pain			0.683
NA	1 (1)	0	
1	2 (2)	83 (83.8)	
5	14 (14.1)	5 (5.1)	
10	82 (82.8)	11 (11.1)	

Abbreviations: CS, cotton swab; n, number; NA, not available; U, unit.

**Table 4:** Intragroup changes pre and postquestionnaire in the Botox 150 U group

Variables	Pretreatment ( *n* = 47)	Posttreatment ( *n* = 47)	*p* -value
Finger penetration anxiety			0.037
1	0	32 (68.1)	
5	15 (31.9)	10 (21.3)	
10	32 (68.1)	5 (10.6)	
Finger penetration pain			0.343
1	0	42 (89.4)	
5	13 (27.7)	4 (8.5)	
10	34 (72.3)	1 (2.1)	
Dilator anxiety			0.000
NA	7 (14.9)	0	
1	0	29 (61.7)	
5	13 (27.7)	11 (23.4)	
10	27 (57.4)	7 (14.9)	
Dilator pain			0.001
NA	7 (14.9)	0	
1	0	42 (89.4)	
5	14 (29.8)	1 (2.1)	
10	26 (55.3)	4 (8.5)	
CS anxiety			not computed
1	22 (46.8)	47 (100)	
5	9 (19.1)	0	
10	16 (34)	0	
CS pain			Not computed
NA	1 (2.1)	0	
1	38 (80.9)	47 (100)	
5	4 (8.5)	0	
10	4 (8.5)	0	
Intercourse anxiety			0.035
1	0	30 (63.8)	
5	13 (27.7)	10 (21.3)	
10	34 (72.3)	7 (14.9)	
Intercourse pain			0.372
1	0	39 (83)	
5	8 (17)	1 (2.1)	
10	39 (83)	7 (14.9)	

Abbreviations: CS, cotton swab; n, number; NA, not available; U, unit.

**Table 5:** Intragroup changes in the Botox 200 U group ( *n* = 52)

Variable	Pretreatment ( *n* = 52)	Posttreatment ( *n* = 52)	*p* -value
Finger penetration anxiety			
1	0	31 (59.6)	0.02
5	14 (26.9)	17 (32.7)	
10	38 (73.1)	4 (7.7)	
Finger penetration pain			0.147
1	0	48 (92.3)	
5	17 (32.7)	4 (7.7)	
10	35 (67.3)	0	
Dilator anxiety			0.000
NA	4 (7.7)	0	
1	0	26 (50)	
5	14 (26.9)	23 (44.2)	
10	34 (65.4)	3 (5.8)	
Dilator pain			0.749
NA	4 (7.7)	0	
1	3 (5.8)	45 (86.5)	
5	11 (21.2)	7 (13.5)	
10	34 (65.4)	0	
CS anxiety			0.592
1	9 (17.3)	48 (92.3)	
5	25 (48.1)	4 (7.7)	
10	18 (34.6)	0	
CS pain			Not computed
1	37 (71.2)	52 (100)	
5	10 (19.2)	0	
10	5 (9.6)	0	
Intercourse anxiety			0.031
NA	1 (1.9)	0	
1	0	32 (61.5)	
5	18 (34.6)	16 (30.8)	
10	33 (63.5)	4 (7.7)	
Intercourse pain			0.922
NA	1 (1.9)	0	
1	2 (3.8)	44 (84.6)	
5	6 (11.5)	4 (7.7)	
10	43 (82.7)	4 (7.7)	

Abbreviations: CS, cotton swab; n, number; NA, not available; U, unit.

Factors like occupation ( *p* = 0.351), residency ( *p* = 0.087), type of vaginismus ( *p* = 0.297) and type of anesthesia ( *p* = 0.496) did not significantly affect the overall outcome of the study cohort, and in individual groups ( *p* > 0.05, data not shown).

## Discussion

No significant difference was found in comparing low-dose Botox (150 U) versus high dose Botox injections (200 U) in vaginismus patients. However, it is estimated that it affected 57% of women in childbearing years in some studies. 8 Overall, patients' mean (SD) age in our study was 30.2 (8.3) and similar between both groups. The mean age of vaginismus patients was in accordance with a study by Yaraghi et al. 4 (28.8–30.8 years) and slightly higher than the mean age of patients (21–28 years) reported in the literature. 3 10 11 The difference could be due to late marriages and cultural taboos; women do not openly speak up about sexual problems. According to the previous studies, the self-reported duration of vaginismus ranges from 3 months to 44 years. 3 15 16 17

In our study, the mean duration of vaginismus was 19.3 months (range: < 1–96 months). The shorter duration of our study could result from religious and cultural norms where procreation is considered an important aspect; many women approached marriage at an earlier stage. 16 Patients who never experienced non-painful intercourse comprise the primary vaginismus group; those who acquired vaginismus later in life are categorized as secondary vaginismus. In our study, the frequency of primary vaginismus (88.9%) was higher than secondary vaginismus (11.1%). The findings are in accordance with previous studies. 15 17 Similar to the study by Velayati et al. 3 (88.6%) ∼ 90% of patients were nulliparous in our study.

Based on the FSFI questionnaire, more than half of patients had anxiety and pain score of 10, suggesting severe anxiety and impossible or extremely painful, respectively. Sexual and reproductive health is considered the defining factors in the well-being of a person. 18 Inability to have intercourse negatively affects an individual's marital satisfaction, stability, overall health, and quality of life. 19 20 Therefore, treatment of vaginismus at earlier stages of marriage is the need of the hour. Various treatment modalities for vaginismus management include physical therapy, Kegel exercises, counseling, psychotherapy, muscle relaxants and lubricants, vaginal dilators, cognitive-behavioral therapy, topical anesthetics, anti-anxiety medications, use of benzodiazepines, hypnotherapy, and Botox injections. 4 8 10 However, none of the treatment approaches are evaluated based on well-designed studies.

Botox injection effectively treats neuromuscular dysfunctions and has been widely used since the successful treatment of the first case in 1997. 21 Botulinum Toxin is a protein neurotoxin produced by anaerobic sporulated bacteria of the Clostridium genus. 22 It induces peripheral muscle relaxation by degrading SNARE proteins and blocking the release of acetylcholine responsible for neurotransmission of muscle contraction. Additionally, Botulinum A also decreases the substance P and glutamate levels as a part of central sensitization, thereby decreasing pain sensitivity. 23 The muscle inactivation lasts until the formation of new fibrils and reconnection of the neuromuscular junction. 24 The following factors must be considered during the delivery of Botox injections: the amount of Botox injected per muscle and total amount and the number of sites injected. 24 To avoid antibody formation, the dose and frequency of Botox should be minimized. Although the extent of paralysis depends on the amount of toxin injected, the data on the minimum dosage required for vaginismus treatment is unclear. Botox doses ranging from 20 to 500 units per injection are deemed successful in treating vaginismus and pelvic pains. 8 23 25 In a comparative analysis, Ghazizadeh et al. 26 reported that a higher dose of Botox (500 U) is more effective than a lower dose (250 U) of Botox. Our study aimed to compare the efficacy of low-dose Botox (150 U) with higher dose Botox (200 U) in vaginismus patients' treatment outcomes.

Pacik et al. 17 explained a direct relationship between muscular spasms and the severity of vaginismus. Spasm of increased muscle activity in levator ani, puborectalis, and bulbocavernosus muscles is common in severe vaginismus grades. Therefore, similar to Park and Paraiso, 27 in our study, Botox was injected explicitly into and around the bulbospongiosus muscle and the lateral submucosal areas of the introitus and additional injections into levator ani muscle, wherever required. Successful paralysis or spasm of bulbospongiosus muscle is indicative of improvement in vaginismus. 28 According to the manufacturer's instructions, we diluted 100 U of Botox with 2 mL of preservative-free saline. Reports also suggest replacing saline with anesthetic solutions for immediate antinociceptive effects. However, it must be used with caution. 25

The ability to achieve satisfactory intercourse is considered the primary outcome in most studies. 9 10 24 29 30 Based on this, the success rate of botulinum injections in vaginismus ranges from 62 to 100%, 23 and patients achieved satisfactory intercourse on the same day of treatment. 10 In our study, Lamont criteria and psychosexual evaluation using a questionnaire by FSFI were used as indicators to check the efficacy of botulinum toxin in a broader perspective. Similar to Bertolasi et al., 31 we observed significant improvement in finger penetration pain and anxiety scores, dilator anxiety and pain scores, and intercourse anxiety scores in individual treatment groups ( *p* < 0.05). In intergroup comparisons of symptom improvements between 150 U and 200 U Botox, except for dilator pain scores ( *p* = 0.015), the pain and anxiety scores of all other variables in both treatment groups were similar ( *p* > 0.05).

Based on an Iranian study, factors including male partner involvement during treatment and cultural background not influencing the treatment outcome, 32 whereas marital and fertility-related anxiety, family and social pressure, and personal guilt negatively affect the treatment outcome. 33 According to Anğin et al., 34 a positive history of vaginismus within a close family and environment negatively affects the treatment outcome. Additionally, sexual abuse decreases the prognosis. While the sexual health of the male partner does not influence the treatment outcome, it affects the duration of treatment. In our study, occupation, residency, type of vaginismus, and type of anesthesia did not significantly affect the overall study cohort's treatment outcome and individual groups.

Although botulinum toxin is generally safe, the use is contraindicated in patients with local infections, albumin hypersensitivity, neuromuscular and bleeding disorders, and the use of anticoagulants. 23 Adverse events are noted with higher doses that include pain, hematoma and infection at the injection site, urinary and anal incontinence, transient blurred vision, and vaginal dryness. 15 25 35 However, urinary and fecal incontinence are seen in higher doses > 100 U. Rarely, an ischiorectal fossa abscess may also occur. 36 To prevent injection-related adverse effects and complications, guidance techniques like electromyography, electrical stimulation, and ultrasound can be used in an office setting to restrict injections to the muscles and vaginal soft tissue only. In our study, since the clinician was careful not to inject the solution anteriorly or posteriorly, side effects were seen in only 19.2% of patients (urinary incontinence versus anal incontinence; 17.2% versus 2%). The frequency of side effects between the 150 U v and 200 U Botox groups were comparable ( *p* = 0.997).

In a country with strict social and religious norms where sexual problems are considered taboo, the present study is the first of its kind with the hope of saving families from breaking apart due to vaginismus-related consequences. The inclusion of a wide range of patients from within and outside the country was one of the strengths. Although the sample size was small, it seemed sufficient to identify differences in the study outcome. A short follow-up period is one of the study's major limitations, as previous studies have reported recurrences following Botox injections. According to previous research, a placebo had an equal or superior effect to Botox injections. 37 38 In their metanalysis, Weinberger et al. 39 conferred that the placebo effect accounts for 67.7% of outcomes in female sexual dysfunction treatment. The inclusion of a placebo arm will assist in assessing the accurate outcome of the test group. Hence, the inability to include a placebo arm in the study was another limitation. Therefore, further placebo-controlled, prospective randomized trials with controlled questionnaires are essential for vaginismus treatment.

In vaginismus patients, detailed discussions and history are mandatory to categorize a patient under an anatomical or psychological disorder, especially during the first gynecological visits. The botulinum injection is a simple, safe, cost-effective, and rapid treatment that can be performed in an inpatient or outpatient setting; clinicians widely appreciate it. However, the effect lasts for around six months. Based on the evidence, the frequency of patients discontinuing follow-up ranges from 1.2 to 47.8%. 34 Therefore, regular follow-ups may be required to check for recurrences. Severe vaginismus is one of the reasons for unconsummated marriages, and continuous failure to have intercourse secondarily leads to male impotence. 40 The fear and anxiety about sex could be due to a lack of sex education in women with no knowledge of sexual anatomy. 41 Therefore, it is of utmost importance to incorporate sex education into the school curriculum.

Moreover, some studies demonstrated no positive or inferior outcome of Botox in vaginismus as compared with other treatment modalities, including physiotherapy, 4 behavioral sex therapy, 42 and better outcomes with a multimodal approach, like dilatators, physical therapy, psychotherapy, and sacral erector spinae block (ESB). 6 Therefore, pre and postprocedure counseling and longtime follow-up are essential during vaginismus treatment. Along with satisfactory intercourse, domains of sexual function and FSFI must also be considered efficacy endpoints of vaginismus treatment. 4 42

## Conclusion

Our study showed that low-dose Botox (150 U) is as effective as high dose Botox injections (200 U) in vaginismus patients. Therefore, low Botox doses should be preferred over high doses to prevent adverse effects and treatment-related complications. Along with Botox injections, it is paramount to incorporate psychotherapy and counseling in patients, especially those with severe anxiety, and fear of sex and gynecological examination. Further randomized controlled trials, including a placebo group, are warranted to evaluate the efficacy of low-dose Botox.
